# Dosimetric effect of CT contrast agent in CyberKnife treatment plans

**DOI:** 10.1186/1748-717X-8-244

**Published:** 2013-10-18

**Authors:** Hee Jung Kim, Ah Ram Chang, Yang-Kyun Park, Sung-Joon Ye

**Affiliations:** 1 Department of Radiation Oncology, Soonchunhyang University Hospital, Seoul, South Korea; 2 Department of Biomedical Engineering and Institute of Radiation Medicine, Seoul National University, Seoul, South Korea; 3 Department of Radiation Oncology, Seoul National University Hospital, Seoul, South Korea; 4 Department of Transdisciplinary Studies and Advanced Institutes of Convergence Technology, Seoul National University, Suwon, South Korea

**Keywords:** CyberKnife, CT contrast, Small field beam

## Abstract

**Background:**

To investigate the effect of computed tomography (CT) contrast enhancement (CE) on the 3D dose distributions of non-coplanar small field beams in the CyberKnife (CK) treatment planning system (TPS) for the stereotactic ablative radiotherapy (SABR).

**Methods:**

Twenty-two pre-CE CT treatment plans were recruited to this retrospective plan study. Their post-CE CT plans were based on the pre-CE CT plan data and calculated using the same MU and beam paths in either Ray-Tracing or Monte Carlo (MC) algorithms. The differences in the doses of the beam path and the reference point between the pre- and post-CE CT plans were compared. The minimum, maximum, and mean doses in dose-volume histograms (DVHs) of target and organs-at-risk (OARs) were also compared.

**Results:**

The dose differences between the pre- and post-CE plans in a single beam path were less than 1.05% in both calculation algorithms, with respect to the prescription dose. At the center of the target volume, it was 1.9% (maximum 6.2%) in Ray-Tracing and 1.6% (maximum 4.0%) in MC. The CA effect showed on average 1.2% difference in the OAR maximum dose (maximum 7.8% in Ray-Tracing and 7.2% in MC). In the lung cases, the CT CE resulted in a dose difference of 2.4% (from 1.0% to 6.5%) without the calculation algorithm effect (maximum 20.3%).

**Conclusions:**

The CK treatment plan using the post-CE CT generally afforded less than 2% dose differences from the pre-CE CT plan. However, it could be up to 7.8% depending on the target positions in a body and be more than 20% with the calculation algorithms. Thus, the post-CE CT in CK treatment plans should be used with careful consideration for the CA effect, target position, and calculation algorithm factors.

## Background

The dose distributions for modern radiotherapy have been accurately predicted with computed tomography (CT)-based treatment planning systems (TPS). The CT data include the Hounsfield unit (HU) as a linear transformation of the beam attenuation that varies with the electron densities of materials on the beam path. The TPS obtains the relative electron density from the relationship between the linear attenuation coefficients and CT HU values to account for the heterogeneity in the patient body. CT scanning is often undertaken with a contrast agent (CA) that includes high-Z radio-opaque materials. The tissue containing CA attenuates the CT X-rays more than normal, resulting in a higher electron density than the actual density. Thus the CT data taken with CA used in TPS give rise to a higher value of monitor unit (MU) than the CT data taken without CA. As a result, a dose higher than intended may be delivered to the patient when daily treated without CA.

Many previous studies reported the effect of CA on the dose calculation for the conventional Linac–based treatment. In a phantom study, Ramm et al. (2001) found that its effect was on average a 3% difference (max. 10.3%) [1]. In other phantom studies, however, it was less than 7% (Rankine et al., 2008), 5% (Robar et al., 2002), and 2% (Lees et al., 2005) [2-4]. The 3-dimensional conformal radiotherapy (3D CRT)/Intensity modulated radiotherapy (IMRT) treatment planning studies using patient CT data with various targets (brain, head and neck, lung, prostate, and so on) presented less than 3%, which could be clinically acceptable [2,4-13]. A similar result (<1.5%) was obtained in the tomotherapy planning system for head and neck cancer [14]. Linac-based stereotactic radiosurgery/stereotactic body radiotherapy (SRS/SBRT) studies also showed less than 3% difference [5,15]. In a Monte Carlo (MC) study for 6 MV Linac beam using a 25 mm collimator, the CA effect was less than 5% in the flattening filter beam and maximum 10.8% in flattening filter-free (FFF) beam [3].

In contrast to the conventional Linac, CyberKnife^®^ (CK) Robotic Radiosurgery System (Accuray Inc., Sunnyvale, CA, USA) utilizes a 6 MV FFF beam with small circular field collimators of 5 mm to 60 mm, and provides distinct characteristics. Unlike 3D conformal radiation therapy and intensity modulated radiation therapy that use the isocentric and coplanar beams fewer than 10 per plan, CK can use not only the isocentric, but also the anisocentric and non-coplanar beams over 100 per plan so that it makes a steep dose fall-off and outstanding conformity. In addition, the thickness of planning CT slices and the grid size of dose calculation are less than 1.5 mm in CK, which is smaller than those used in most conventional Linac plans. CK has two algorithms for the dose calculation: Ray-Tracing and MC. The MC algorithm has been reported to have a high accuracy to calculate doses in heterogeneous materials [16-18]. Due to the differences of the beam characteristics mentioned before and the calculation algorithms, the previous results of the CA effect on the dose calculation for the conventional Linac may not valid for CK. Moreover the small dose difference in the stereotactic ablative radiotherapy (SABR) using a large dose per fraction can increase the risk of the severe normal tissue complication. To our best knowledge, however, the influence of CA on the CK treatment plans has not been reported yet.

To investigate the effect of CA in CK, two CT data sets (pre-contrast enhanced (CE) and post-CE CTs) were acquired. The post-CE CT was used to fuse and delineate the target and organs at risk (OARs), and the pre-CE CT to calculate doses for treatment plan. Therefore, this study used both CT sets to investigate the dose difference in the CK TPS. The pre- and post-CE CTs were applied to calculate the 3D dose distributions for the same plan beams using the two calculation algorithms. The point dose for each single beam path in the plan and the dose-volume histogram (DVH) of the target and OARs were analyzed.

## Methods

Twenty-two treatment plans were retrospectively recruited in this study, 6 for spine, 5 for prostate, 4 for lung, 2 for liver, 3 for abdominal lymph node, 1 for kidney and 1 for pleura cancers. The institutional review board (IRB NO. 2011-116) for this study was approved.

The pre- and post-CE CT were scanned before and after the CA injection to the patient. The IOMERON 350 (Bracco, Milan, Italy) was used as a CA with a concentration of 350 mg I/ml and a relative density of 1.39. The amount of CA was 140 ml for every patient. The delay time between the CA injection and the start of the CT scan was 70 sec. The CT scanner was SOMATOM Sensation 64-channel (Siemens, Erlangen, Germany). The CT scans were performed with the same position. The time interval between pre- and post-CE CT scans was less than two minutes. Both CT scans were taken at the phase of suspended full expiration. All patients had the CT scans at 120 kVp with 83-250 mAs.

The patients were treated with the G4 CK system and treatment planning was made using a Multi-Plan v2.0 TPS (Accuray Inc., Sunnyvale, CA, USA). A total of 8 collimators from 10 mm to 40 mm were used, but for each patient plan the maximum number of collimator used was 2. On average 209 beams were used in a plan with an average of 183 beams per collimator. The average MU per beam was 260 MU (17 MU ~ 782 MU). In the planning, the maximum MU per beam per fraction was set to the range of 150 MU to 200 MU. The patients were treated with pre-CE CT plans. Their post-CE CT plans were based on the pre-CE CT plan data and calculated using the same MU and beam paths in either Ray-tracing or MC algorithms. Therefore, four plans per patient were generated and their structure and 3D dose distributions were exported as a DICOM file for analysis.

A MIM software (MIM Software Inc., Cleveland, OH, USA) was used to evaluate the amount of HU change in OARs between the pre- and post-CE CTs. The varied electron densities of the voxels in the CT depending on the degree of CA absorption change the effective depths through the beam paths affecting the computation of the dose. The effective depths for small field beams in a plan were calculated by the Multi-Plan TPS. The beam text file ('BM_patient ID_plan name.txt’) included the effective depths for each beam path. The reference point information was also acquired from the same file. The reference point was positioned at the center of the target volume. The differences in effective depths and doses of the beam path and the reference point between the pre- and post-CE CT plans were compared. The number of beams with a dose difference more than the average difference was counted and the percentage of the beams was calculated.

The DVHs of the target and OAR were calculated from the DICOM files of the structures and 3D doses by using the MIM software. The minimum, maximum, and mean doses were also compared. The relative dose difference (%) was defined as follows:

$$\begin{array}{l} \text{Relative}\;\text{dose}\;\text{difference}\;\left[\%\right]\\ \;=\frac{\left|\left(\text{Dose}\;\left[\text{cGy}\right]\;\text{with}\;\text{Pre}\_\text{CE}\;\text{CT}\right)–\left(\text{Dose}\;\left[\text{cGy}\right]\;\text{with}\;\text{Post}\_\text{CE}\;\text{CT}\right)\right|}{\text{Prescription}\;\text{Dose}\;\left[\text{cGy}\right]}. \end{array}$$

The dose differences between the pre- and post-CE CT plans were tested with paired *t*-test. The dose differences between Ray-tracing and MC were also calculated.

## Results

### HU changes due to the CA injection

The degree of HU change for OARs due to the CA injection in CT scans is shown in Table 1. The baselines of HU were the average HU values of OARs in pre-CE CTs. The aorta showed the largest change in HU, followed by the heart and kidney. These 3 CA-sensitive OARs showed an average difference of over 124 HU (maximum 223).

**Table 1:** HU changes of OARs due to the CA injection

**OARs**	**No. of cases**	**Baseline [HU]**	**Max changes [HU]**	**Average changes [HU]**
Aorta	5	53	223	171 ± 40
Heart	5	12	164	155 ± 7
Kidney	10	13	158	124 ± 33
Lung	14	-650	121	45 ± 32
Liver	12	40	64	45 ± 14
Stomach	3	-64	55	40 ± 16
Rectum	6	17	37	33 ± 5
Urethra	5	37	42	32 ± 9
Esophagus	14	-126	67	31 ± 14
PTV	22	-16	113	30 ± 28
Bowel	17	-72	40	27 ± 9
Bladder	6	11	14	8 ± 3
Spinal cord/cauda equina	17	41	12	6 ± 3
Femur head	5	234	4	2 ± 1

### Effect of contrast enhancement (CE) on the effective depth, target volume and dose of a single beam path

With the CA, the effective depths increased, resulting in decreased doses. Table 2 shows that the change in the effective depth was 4.0 mm on average, with a maximum of 68.1 mm. We analyzed the correlation between the target volumes and the dosimetric differences and found no significant correlation between them in various aspects, dose maximum (P-value = 0.08 and 0.07 in Ray-tracing and MC), mean dose (P-value = 0.09 and 0.08 in Ray-tracing and MC), and dose minimum (P-value = 0.11 and 0.33 in Ray-tracing and MC). As shown in Table 3, the dose differences between the pre- and post-CE plans in a single beam path were insignificant (in average, 0.01 ± 0.03% and 0.05 ± 0.09% of the prescription dose for the Ray-tracing and MC algorithms, respectively). But it was nevertheless statistically significant (P-value < 0.001).

**Table 2:** Difference of effective depth between pre- and post-CE

	**Effective depth difference |(pre-CE) – (post-CE)|**
Max	68.1 mm
Average	4.0 mm
1 SD	5.1 mm

**Table 3:** Dose difference of single beam paths between pre- and post-CE

	**Relative dose difference |(pre-CE) – (post-CE)|/Prescription dose**	
	**RT**	**MC**
Max	0.49%	1.05%
Average	0.01%	0.05%
1 SD	0.03%	0.09%

### Effect of contrast enhancement (CE) on doses and DVH of target and OAR

Table 4 shows lower doses with same MU at the reference point due to the existence of CA in both algorithms. Due to the CE, the dose difference at the center of the target volume (i.e., the reference point) was 1.9% ± 1.6% (maximum 6.2%) in Ray-tracing and 1.6% ± 1.1% (maximum 4.0%) in MC; this difference was statistically significant (P-value < 0.001). It was maximum 409 cGy under the prescription dose of 6600 cGy. The highest dose difference occurred in cases 1 and 2, the plan for lung (6.2% in Ray-tracing) and pleura (4.0% in MC). For the pelvis region (cases 14, 16, and 19-22), the average dose difference at the reference point was 0.7% in Ray-tracing and 0.8% in MC. For other regions, it was 2.3% and 1.9% in Ray-tracing and MC, respectively.

**Table 4:** Reference point dose difference between pre- and post-CE, compared with the average change of effective depth

**Case No.**	**Target**	**Mean d**_**eff **_**difference**	**Reference point dose**	
			**In RT**	**In MC**
1	Lung (Left Lower Lobe)	11.4 mm	6.2%	3.8%
2	Lt. Pleura	11.0 mm	5.7%	4.0%
3	Lung (Left Upper Lobe)	7.4 mm	3.3%	2.4%
4	T4	6.4 mm	2.7%	3.8%
5	Liver (Segment 6)	4.7 mm	2.7%	3.0%
6	PAN^*^	5.2 mm	2.4%	0.2%
7	Lung (Left Upper Lobe)	4.1 mm	2.1%	1.7%
8	L2	3.9 mm	2.1%	1.9%
9	SMA LN^†^	3.7 mm	1.9%	1.7%
10	Liver (Segment 8)	3.2 mm	1.6%	1.1%
11	T1-2^‡^	3.1 mm	1.5%	1.0%
12	L2-5^§^	3.6 mm	1.3%	2.1%
13	Lung (Right Middle Lobe)	2.6 mm	1.2%	1.3%
14	Iliac LN	2.0 mm	1.1%	0.6%
15	Lt. Kidney (Renal Pelvis)	4.4 mm	1.0%	1.2%
16	Prostate	1.2 mm	0.8%	1.1%
17	C5^||^	1.5 mm	0.7%	1.1%
18	C5-T2	3.1 mm	0.6%	0.5%
19	Prostate	1.2 mm	0.6%	0.7%
20	Prostate	1.6 mm	0.6%	1.1%
21	Prostate	1.1 mm	0.5%	0.4%
22	Prostate	1.1 mm	0.4%	0.9%
	Average ± 1SD	4.0 ± 2.9 mm	1.9 ± 1.6%	1.6 ± 1.1%

^*^PAN : Paraaortic lymph node.

^†^SMA LN : Superior mesenteric artery lymph node.

^‡^T : Thoracic spine.

^§^L : Lumbar spine.

^||^C : Cervical spine.

When the mean difference of effective depths had a difference over 10 mm, the dose difference at the target center (e.g., the reference point) was 4.9% on average considering both algorithms together. However, it was 1.1% when the mean d_eff_ difference was less than 4 mm.

**Figure 1:**
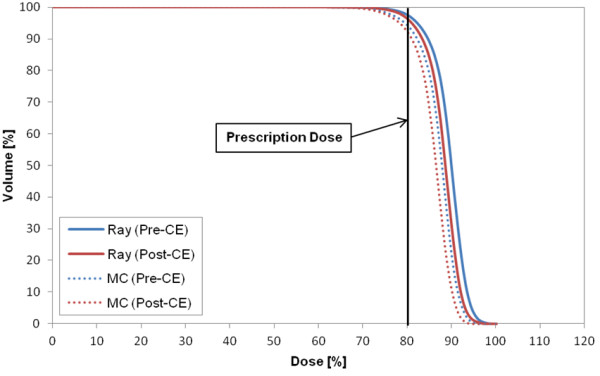
DVH of target in case 8 (L-spine (L2) plan), as an example to show the CA effect on the target in most of the cases.

**Figure 2:**
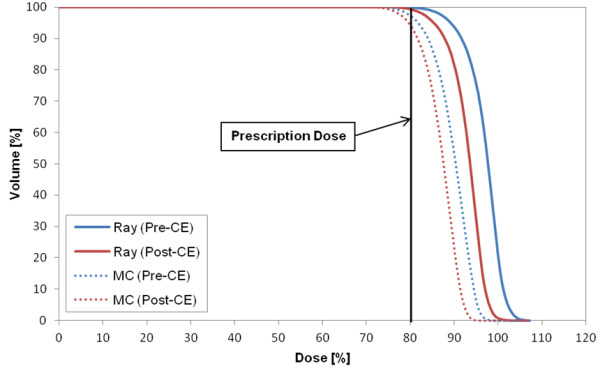
DVH of target in case 2 (pleura plan) that shows the largest difference in the target dose between the two CTs (pre- and post-CE CTs).

**Figure 3:**
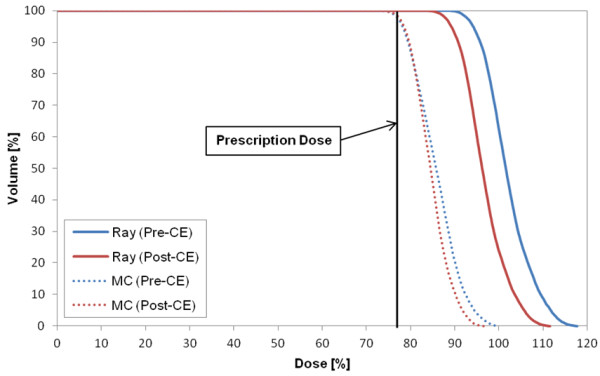
DVH of target in case 1 (lung (LLL) plan) that is a specific case to show the large dose difference due to the combined effect of the contrast enhancement (CE) and different calculation algorithms.

Figure 1 shows the DVH of target in the plan for case 8 that represents the CA effect on the target in most cases. The target volume receiving the prescription dose was changed by 2% in Ray-tracing and 3.4% in MC. The largest difference in the target between the two CTs (p- and post-CE CTs) occurred in case 2 (pleura plan) (Figure 2). As a specific case, the target DVH for case 1 [lung (LLL) plan] in Figure 3 showed a large dose difference especially in Ray-tracing due to the combined effect of CE and algorithm limitation. Table 5 shows that the differences in the minimum, maximum and mean doses to the target were 1.0% to 1.9% on average, and were statistically significant (P-value <0.05).

**Table 5:** Target dose difference between pre- and post-CE

	**In RT**			**In MC**		
	**D**_**min**_	**D**_**max**_	**D**_**mean**_	**D**_**min**_	**D**_**max**_	**D**_**mean**_
Max	5.3%	7.8%	6.8%	3.2%	4.5%	3.4%
Average	1.3%	1.9%	1.7%	1.0%	1.6%	1.2%
1 SD	1.3%	1.8%	1.6%	0.8%	1.1%	0.7%
p-value	0.011	0.005	0.002	0.012	<0.001	<0.001

**Figure 4:**
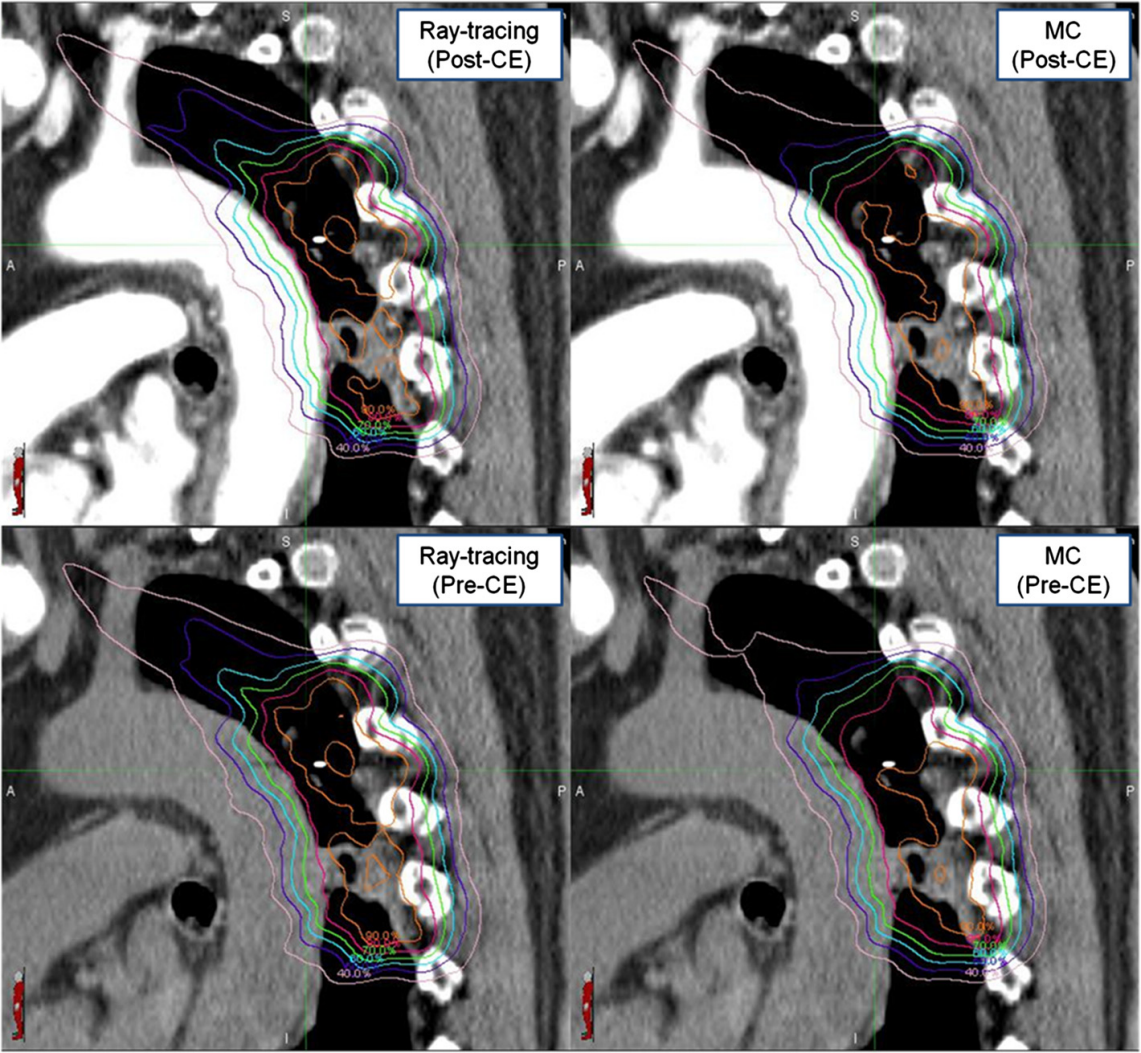
Dose distribution in case 2 having a maximum difference in mean dose to the target, depending on the CA existence and the different calculation algorithms.

In Table 6, the minimum and mean doses differences of the OAR were less than 0.3% on average. The maximum dose of contrast-enhanced OAR was decreased by 0.8% ± 1.1% (P-value < 0.001) in Ray-tracing and 0.9% ± 1.2% (P-value < 0.001) in MC. The maximum OAR dose difference in Tables 6 and 7 was found in case 1. The maximum difference in the mean dose was shown in case 2. For this case, Figure 4 shows the dose distributions depending on the CA existence and the different calculation algorithms.

**Table 6:** OAR dose difference between pre- and post-CE

	**In RT**			**In MC**		
	**D**_**min**_	**D**_**max**_	**D**_**mean**_	**D**_**min**_	**D**_**max**_	**D**_**mean**_
Max	2.7%	7.8%	1.4%	1.3%	7.2%	2.1%
Average	0.1%	0.8%	0.2%	0.1%	0.9%	0.2%
1 SD	0.2%	1.1%	0.2%	0.2%	1.2%	0.4%
p-value	0.727	<0.001	<0.001	0.029	<0.001	<0.001

**Table 7:** Maximum/Mean values of maximum-dose difference in each OAR

**OARs**	**Maximum of D**_**max**_		**Average of D**_**max**_	
	**In RT**	**In MC**	**In RT**	**In MC**
Aorta	3.8%	4.5%	2.0%	2.8%
Heart	3.5%	3.3%	1.3%	1.5%
Lung	7.8%	5.9%	1.5%	1.3%
Liver	2.9%	7.2%	0.6%	1.4%
Kidney	2.4%	2.7%	0.7%	0.8%
Bowel	1.7%	2.5%	0.7%	0.8%
Spinal cord/Cauda equina	2.0%	1.8%	0.7%	0.7%
Urethra	0.6%	1.1%	0.4%	0.6%
Rectum	0.7%	1.2%	0.5%	0.5%
Esophagus	2.0%	1.6%	0.7%	0.5%
Stomach	0.2%	0.9%	0.1%	0.4%
Bladder	0.8%	0.7%	0.4%	0.3%
Femur head	0.3%	0.6%	0.1%	0.2%

**Figure 5:**
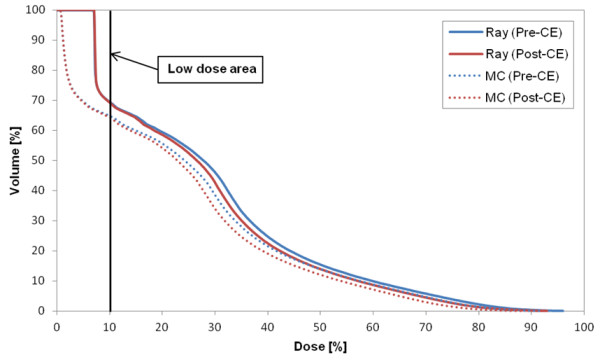
DVH of aorta in case 2 (pleura plan).

**Figure 6:**
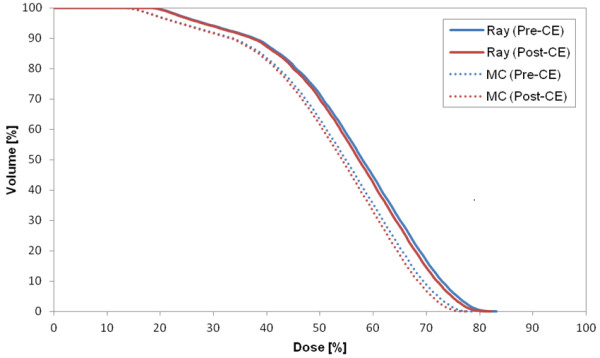
DVH of cauda equina in case 12 (L-spine (L2 to L5) plan).

Table 7 lists the maximum and mean values of the maximum-dose difference due to the CE on OARs. Among the OARs, the lung had the largest differences in the maximum dose (7.8% in Ray-tracing). In MC, the highest difference in the mean value occurred in aorta (2.8%), followed by heart (1.5%), liver (1.4%), lung (1.3%), and kidney (0.8%). The maximum difference of maximum dose of these OARs was over 2.4%. Figures 5 and 6 show the DVHs of aorta and cauda equina, as examples of the largest maximum-dose difference and the normal-dose difference.

### Dose differences due to different calculation algorithms

The doses calculated with the two different algorithms (Ray-tracing and MC) were compared. For small field beams, the dose difference between the two calculation algorithms was on average 0.1% (maximum 2% or 100 cGy) per beam, but it was statistically significant (P-value < 0.001). The reference point dose difference (average 2.6%) between the two algorithms was not statistically significant. However, it was maximum 8.7%, depending on the position of target.

The difference in the target doses between the two algorithms was on average 3.5% ± 4.1% (maximum 20.3%) (P-value = 0.013). However, for the OARs, the mean dose difference was statistically significant (average 4.3% ± 2.6%, maximum 13.3%). In low doses (less than 10% of the prescription dose), a large dose difference between the algorithms is shown in Figure 5.

## Discussion

The dose at the center of the target had a difference of 1.9% in Ray-tracing and 1.6% in MC because the CK treatment plans normally used over 200 beams, although the dose difference due to CA was small (on average 0.1% or less for the prescription dose) for each single beam path with small field size in a plan. In CK treatment planning, the dose difference between MC and Ray-tracing due to the CA is resulted from the various planning parameters including the MU (0-150 MU in this study) of single beam path, the distance between the central axis of beam and the reference point, the collimator size, the number of beams passing the CA-uptaken structure, and the calculation accuracy of scattered dose.

The study from Amato, et al. showed that the relative increase of HU due to CA was 74% (from 121.7 to 161.9 HU) for kidney, 48% (from 95.8 to 105.8 HU) for thyroid, 22% (from 55.3 to 62.2 HU) for liver, and so on [19]. In addition, in this study, the HU and dose to the aorta were affected most significantly by CE, followed in order by the heart, liver, lung, and kidney. Those OARs sensitive to CE had a difference of 2.8% in the average of differences of maximum-dose. Since many beams in the pleura, lung and thoracic spine plans passed through the aorta, lung and heart, those plans had an increased difference in the mean dose to the target, which was 5.1% and 3.4% in the pleura plan, 3.3% and 1.3% in the lung plan and 2.6% and 2.1% in the thoracic spine plan with Ray-tracing and MC, respectively. The maximum dose in the lung, aorta, heart and spinal cord was also reduced by up to 7.8% due to the CE. From these observations, for a target close to OARs that increased the CA absorption, the CK treatment plan using the post-CE CT could deliver a higher dose to the OAR as well as to the target. This result can lead to the severe OAR complication considering a large dose per fraction of SABR. Unlike the other commercial TPS for Linac, the Multi-Plan v2.0 TPS of CK cannot override the density for the structures. The fine-tuning of MU per single beam path in a plan can modify the dose distribution but the re-optimization only can arrange the beam paths to control the entrance of beams into the CA-uptaken structures. Therefore, the post-CE CT should be carefully used in CK treatment planning if a target is positioned where many beams pass through the aorta or lung, because a dose difference can occur due to the CE. On the other hand, when the target is in the pelvis region (e.g., prostate, iliac lymph node, and lumbar spine) or only a few beams pass the aorta or lung, use of the post-CE CT in CK planning is clinically acceptable (≤1%).

For the dose calculation, the Ray-tracing algorithm uses the commissioned beam data measured in water and the effective depth and the MC uses the electron transport algorithm calculating the absorbed dose to various materials that can provide the higher accuracy on the target dose calculation in a patient than the Ray-tracing algorithm. The dose distribution for the lung plan differed by up to 28% depending on the calculation algorithm (Ray-tracing or MC) [20]. In this study, the maximum dose difference for the lung plan was 20.3% in the target and 13.3% in the OARs. According to this study and previous literatures [16-18,20], recommended is the use of MC algorithm to calculate the target dose for the SABR CK planning in lung cancer treatment or in other lesions using the large number of beams passing through the lung. It is noted that the MC algorithm results in the large statistical uncertainty for the low dose region. In addition, the CT CE in the lung cases resulted in a dose difference of 2.4% (from 1.0% to 6.5%) without this calculation algorithm effect. Thus, the use of both the post-CE CT and Ray-tracing algorithm can lead to an incorrect dose calculation and an inappropriate CK plan for lung cancer patient treatment.

## Conclusions

The CK treatment plan showed a mean dose difference of less than 2% in both the target and OARs due to the CT CE, although the CK utilizes the small field collimators, FFF beams, and multiple non-coplanar beams more than 200. However it could be up to 7.8% to the target and OARs, depending on the target position in a body. In addition to the use of CA, the dose difference can make a large difference of up to 20% with calculation–algorithm effect combined. The large dose difference can lead to the severe complication to OAR in SABR considering a high dose per fraction. In conclusion, the post-CE CT in CK treatment planning should be used with careful consideration for these factors when the target is close to CA-sensitive OARs.

## Abbreviations

CT: Computed tomography; TPS: Treatment planning systems; OAR: Organs-at-risk; HU: Hounsfield unit; CA: Contrast agent; MU: Monitor unit; 3D CRT: 3-dimensional conformal radiotherapy; IMRT: Intensity modulated radiotherapy; SRS: Stereotactic radiosurgery; SBRT: Stereotactic body radiotherapy; MC: Monte Carlo; FFF: Flattening filter-free; CK: CyberKnife; SABR: Stereotactic ablative radiotherapy; CE: Contrast enhanced; DVH: Dose-volume histogram.

## Competing interests

The authors declare that they have no competing interests.

## Authors’ contributions

HJK designed the study, acquired and interpreted the data, and wrote the manuscript. ARJ contributed to the interpretation of the data and involved in drafting the manuscript. YKP participated to data analysis. SJY contributed to the overall experimental design and involved in revising the manuscript critically. All authors have given final approval of the version to be published.

## References

[B1] Ramm U Damrau M Mose S Manegold KH Rahl CG Bottcher HD Influence of CT contrast agents on dose calculations in a 3D treatment planning system Phys Med Biol 2001 46 2631 2635 10.1088/0031-9155/46/10/308 11686279

[B2] Rankine AW Lanzon PJ Spry NA Effect of contrast media on megavoltage photon beam dosimetry Med Dosim 2008 33 169 174 10.1016/j.meddos.2007.04.007 18674680

[B3] Robar JL Riccio SA Martin MA Tumour dose enhancement using modified megavoltage photon beams and contrast media Phys Med Biol 2002 47 2433 2449 10.1088/0031-9155/47/14/305 12171332

[B4] Lees J Holloway L Fuller M Forstner D Effect of intravenous contrast on treatment planning system dose calculations in the lung Australas Phys Eng Sci Med 2005 28 190 195 10.1007/BF03178715 16250475

[B5] Xiao J Zhang H Gong Y Fu Y Tang B Wang S Jiang Q Li P Feasibility of using intravenous contrast-enhanced computed tomography (CT) scans in lung cancer treatment planning Radiother Oncol 2010 96 73 77 10.1016/j.radonc.2010.02.029 20347496

[B6] Weber DC Rouzaud M Miralbell R Bladder opacification does not significantly influence dose distribution in conformal radiotherapy of prostate cancer Radiother Oncol 2001 59 95 97 10.1016/S0167-8140(01)00306-1 11295212

[B7] Shibamoto Y Naruse A Fukuma H Ayakawa S Sugie C Tomita N Influence of contrast materials on dose calculation in radiotherapy planning using computed tomography for tumors at various anatomical regions: a prospective study Radiother Oncol 2007 84 52 55 10.1016/j.radonc.2007.05.015 17532496

[B8] Shi W Liu C Lu B Yeung A Newlin HE Amdur RJ Olivier KR The effect of intravenous contrast on photon radiation therapy dose calculations for lung cancer Am J Clin Oncol 2010 33 153 156 19806038 10.1097/COC.0b013e3181a44637

[B9] Liauw SL Amdur RJ Mendenhall WM Palta J Kim S The effect of intravenous contrast on intensity-modulated radiation therapy dose calculations for head and neck cancer Am J Clin Oncol 2005 28 456 459 10.1097/01.coc.0000170796.89560.02 16199983

[B10] Elawadi A-S Bayoumi Y Alomran R El-Moniem RA Ismail A Alamro A Does intravenous contrast agent affect dose calculations of three dimensional treatment planning system? Bull Alex Fac Med 2009 45 103 108

[B11] Choi Y Kim JK Lee HS Hur WJ Hong YS Park S Ahn K Cho H Influence of intravenous contrast agent on dose calculations of intensity modulated radiation therapy plans for head and neck cancer Radiother Oncol 2006 81 158 162 10.1016/j.radonc.2006.09.010 17050020

[B12] Burridge NA Rowbottom CG Burt PA Effect of contrast enhanced CT scans on heterogeneity corrected dose computations in the lung J Appl Clin Med Phys 2006 7 1 12 10.1120/jacmp.2027.25366 17533351 PMC5722395

[B13] Xiong W Huang D Gewanter R Burman C SU-E-T-545: dose comparison between intravenous contrast-enhanced CT and non contrast CT in treatment planning AAPM 2012 3831 10.1118/1.4735634 28518515

[B14] Xu Y Watchman C Krafft S Jang S SU-GG-T-607: effect of CT contrast materials on dose calculations in tomotherapy planning system AAPM 2010 3327

[B15] Zabel-du Bois A Ackermann B Hauswald H Schramm O Sroka-Perez G Huber P Debus J Milker-Zabel S Influence of intravenous contrast agent on dose calculation in 3-D treatment planning for radiosurgery of cerebral arteriovenous malformations Strahlenther Onkol 2009 185 318 324 10.1007/s00066-009-1927-6 19440671

[B16] Ma CM Li JS Deng J Fan J Implementation of Monte Carlo Dose calculation for CyberKnife treatment planning J Phys 2008 102 012016

[B17] Wilcox EE Daskalov GM Accuracy of dose measurements and calculations within and beyond heterogeneous tissues for 6 MV photon fields smaller than 4 cm produced by Cyberknife Med Phys 2008 35 2259 10.1118/1.2912179 18649456

[B18] Ma C Li J Deng J Fan J WE-C-AUD-03: Investigation of Fast Monte Carlo Dose Calculation for CyberKnife SRS/SRT Treatment Planning Med Phys 2007 34 2589 2590

[B19] Amato E Lizio D Settineri N Di Pasquale A Salamone I Pandolfo I A method to evaluate the dose increase in CT with iodinated contrast medium Med Phys 2010 37 4249 4256 10.1118/1.3460797 20879585

[B20] Sharma SC Ott JT Williams JB Dickow D Clinical implications of adopting Monte Carlo treatment planning for CyberKnife J Appl Clin Med Phys 2010 11 3142 20160699 10.1120/jacmp.v11i1.3142 PMC5719782

